# *Coprosma kawaikiniensis* (Rubiaceae) a new species from the *Dubautia*-*Sadleria* shrubland-fernland community on Kaua‘i, Hawaiian Islands

**DOI:** 10.3897/phytokeys.60.6406

**Published:** 2016-02-11

**Authors:** Kenneth R. Wood, David H. Lorence, Michael Kiehn

**Affiliations:** 1 National Tropical Botanical Garden, 3530 Papalina Road, Kalaheo, HI 96741, USA; 2 Department of Botany and Biodiversity Research and Core Facility Botanical Garden, University of Vienna, Rennweg 14, 1030 Vienna, Austria

**Keywords:** Rubiaceae, *Coprosma*, new species, *Dubautia*-*Sadleria* shrubland-fernland, Hawaiian Islands, Kaua‘i, high endemism, Critically Endangered

## Abstract

*Coprosma kawaikiniensis* K.R. Wood, Lorence & Kiehn (Rubiaceae), a rare endemic tree from Kaua‘i, Hawaiian Islands, is described and illustrated along with a previously undescribed endemic plant community, the *Dubautia*-*Sadleria* shrubland-fernland (DSSF). The new species differs from Hawai‘i congeners by its combination of opposite, long, elliptic to narrowly elliptic or ovate-elliptic leaves with revolute margins; caducous stipules 7–10 mm long, externally glabrous, densely hirtellous-pilose near the margins of the inner surface; unbranched inflorescences with peduncles 20–28 mm long; flowers 6–8 per cluster; and persistent calyx tube with 4–8 irregular dentate lobes. Known only from the windward slopes and ridges of southeastern Kaua‘i below the Kawaikini summit, *Coprosma kawaikiniensis* falls into the IUCN Critically Endangered (CR) Red List category.

## Introduction

*Coprosma* J.R.Forst. & G.Forst., in the family Rubiaceae, is a genus of approximately 110 species of dioecious wind-pollinated shrubs or small trees widely distributed on Pacific islands, with a primary center of diversity in New Zealand (ca. 50 spp.), and secondary centers of diversity in the Hawaiian Islands (13 spp.), New Guinea (11 spp.), and Australia (8 spp.) (Wagner and Lorence 2011; Cantley et al. 2014). The remaining species are scattered over a wide area of the Pacific Basin, extending from Borneo and Java to Rapa Nui in southeastern Polynesia, and the Juan Fernández Islands (Smith 1988). There are six endemic *Coprosma* in the Marquesas Islands including three newly described by Wagner and Lorence (2011), four in the Society Islands (Welsh 1998), three in the Australs, two in Samoa, and one each in the Tuamotu Islands, Pitcairn Island, and Cook Islands.

Oliver (1935) divided the genus into seven groups, most of which were subdivided into smaller groups of presumably closely related species. He placed all southeastern Polynesian species then known into his *Coprosma pyrifolia* (Hook. & Arn.) Skottsb. group characterized by relatively large, usually obovate to ovate leaves with finely reticulate venation, entire to denticulate triangular stipules, male flowers in small clusters with a calyx present, and three female flowers per cluster, the calyx lobes as long as or shorter than the tube, and fruit red or orange. He hypothesized that this group was related to similar species in New Zealand. Florence (1986) described two new Marquesan species and suggested they and the one other known Marquesan species were allied with the orange-fruited Hawaiian species. Although Heads (1996) included no Polynesian *Coprosma* species in his sparse sampling of the genus, he supported Florence’s hypothesis by placing the three known Marquesan species in a group along with the Hawaiian species, rather than the *Coprosma pyrifolia* group where all of the other southeastern Polynesian species were placed. A molecular study of Tribe Anthospermeae (Anderson et al. 2001), in which 6 of 16 of the taxonomic groups recognized by Heads (1996) were sampled, indicates an apparent Australian origin of *Coprosma* and possible independent colonization of Fiji and Hawaiian Islands from New Zealand. Based on molecular phylogenetic analyses of ITS and ETS regions Cantley et al. (2014) provided new biogeographic insights into Pacific *Coprosma* species. Their analyses suggest two independent colonizations of *Coprosma* to the Hawaiian Islands. The majority (12) of the 13 Hawaiian species form a monophyletic group closely related to red- and orange-fruited species from the Marquesas and Austral Islands, whereas the single black-fruited species (*Coprosma ernodeoides* A.Gray) represents a separate colonization to Hawai‘i from an unknown origin, perhaps New Zealand or Tasmania (Wagner and Lorence 2011). This view is also corroborated by the fact that *Coprosma ernodeoides* is a very high polyploid (2n ≥ 220), whereas all other cytologically investigated Hawaiian taxa are tetraploids with 2n = 44 chromosomes (Kiehn 2005).The discovery and publication of *Coprosma kawaikiniensis*, a member of the red- and orange-fruited group, now brings the number of Hawaiian *Coprosma* species to 14.

## Methods

All measurements and descriptions were taken from dried herbarium specimens or from notes made in the field and are presented in the descriptions as follows: length × width, followed by units of measurement (mm or cm).

## Taxonomic treatment

### Coprosma kawaikiniensis

Taxon classificationPlantaeGentianalesRubiaceae

K.R. Wood, Lorence & Kiehn sp. nov.

urn:lsid:ipni.org:names:77152891-1

Figs 1 , 5

#### Diagnosis.

Differs from Hawai‘i congeners by its combination of opposite, long, elliptic to narrowly elliptic or ovate-elliptic leaves with revolute margins; caducous stipules 7–10 mm long, externally glabrous with dense hirtellous-pilose hairs near the margins of the inner surface; unbranched inflorescences with peduncles 20–28 mm long; flowers 6–8 per cluster; and persistent calyx tube with 4–8 irregular dentate lobes.

#### Type.

**USA. Hawai‘i. Kaua‘i**: Lihu‘e District, ridge below Kawaikini, Ioli headwaters, 26 Aug 1999, *K.R. Wood & M. Query 7978*, (holotype: PTBG-050238; isotypes (to be distributed): BISH, MO, P, US, WU).

#### Description.

***Trees*** 2–4 m tall, dioecious, bark grey-brown, trunk with branches or knobby protuberances; branchlets glabrous, light brown, 3–4 mm diam., nodose with leaf and stipule scars. ***Leaves*** opposite, decussate, glabrous, blades elliptic to narrowly elliptic or ovate-elliptic, 40–76 ×11–22(–26) mm, pinnately veined with 8–10 pairs of secondary veins, higher level venation conspicuously reticulate, occasionally impressed adaxially, small pit domatia often present located abaxially along midrib near juncture with secondary veins, sometimes absent, margins slightly revolute, apex acute, base attenuate and decurrent; petioles 5–11 mm long, narrowly winged distally, purple-brown; stipules 7–10 mm long, connate for 1/3–1/2 of their length into a cylindrical sheath (1–)2–4 mm long, free apical portion (3–)4–6 mm long, acuminate to a conspicuous, thickly carinate-subulate apex with a claw-like appendage, externally glabrous, internally densely hirtellous-pilose distally, sheath margins with shortly ciliate fringe and sometimes a few short callous protuberances or glandular appendages 0.1–0.2 mm long, as long as the ciliate hairs. ***Inflorescences*** axillary, mostly simple and capituliform, subtended by a pair of connate, broadly ovate bracts, 6–8-flowered, peduncles 20–35 mm long, or rarely bearing an additional peduncle with one additional pair of subsessile, 3–5-flowered cymules subtended by connate, broadly ovate bracts 2–3 × 2–3 mm with ciliate margins. ***Flowers***: staminate flowers mostly in groups of 6–8 on peduncles 20–28 × 0.7–0.8 mm, subtended by a pair of connate, broadly ovate bracts 2–3 × 2–3 mm with ciliate margins; flowers subsessile, the calyx cup-shaped, irregularly (4–)8-lobed, cup 0.8–1 mm long, the lobes 1–2.2 × 0.8–1.5 mm, apex entire or irregularly 2–4-dentate, glabrous or sparsely hirtellous; corolla (only seen in bud) glabrous, 6–6.5 mm long, the tube 2–3 mm long, 4–5-lobed, the lobes 3.5–4 mm long, stamens 8, the staminal filaments 1.5–2 mm long, the anthers 3.6–4 mm long, apex acuminate, base sagittate, the pistillode 2.5 mm long. Female flowers and inflorescence not seen. ***Infructescences*** with peduncle 20–28 mm long, flattened, fruits in terminal cluster of 3–6 subtended by cupuliform pair of connate bracts 2–3 × 2–2.5 mm, margins ciliolate; fruits orange when fresh, broadly ellipsoid to broadly obovoid, 5.5–6 × 4.5–5 mm, glabrous, surface drying wrinkled, weakly 4-ribbed, apex with persistent irregularly lobed calyx 1.5 mm long. ***Pyrenes*** 2, broadly ellipsoid to broadly obovoid, plano-convex, 5.7–6 × 4.1–4.5 mm, brown, with low dorsal ridge in distal 1/3–1/2.

**Figure 1.**
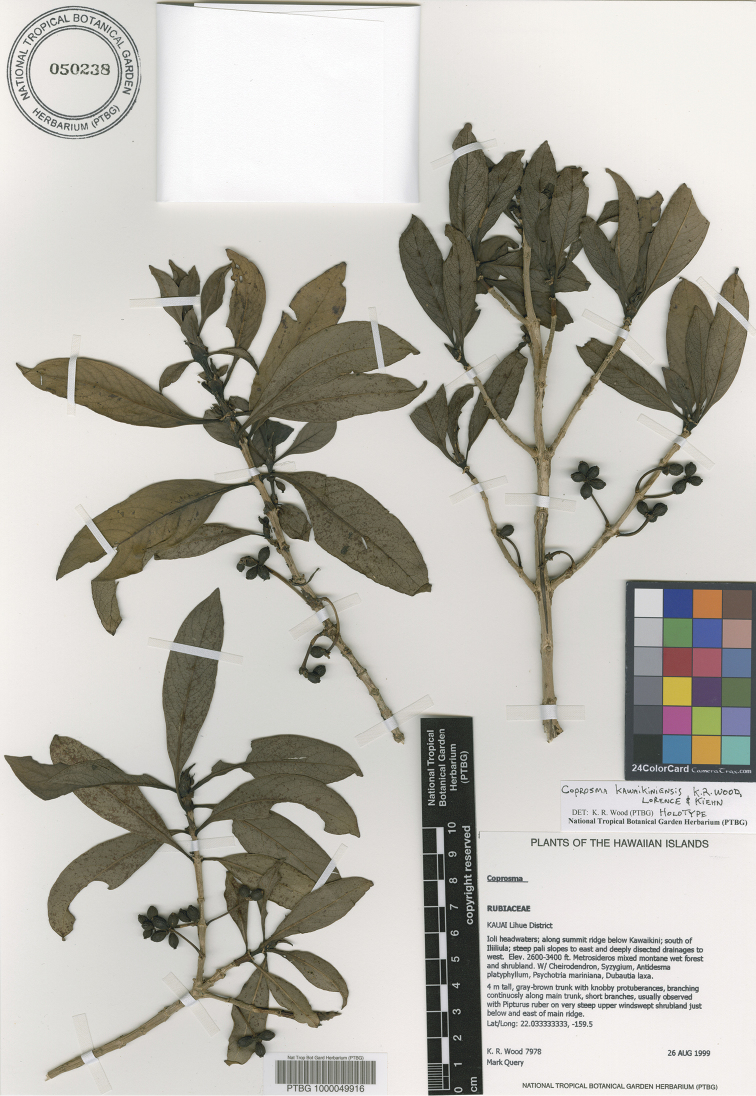
*Coprosma kawaikiniensis* K.R.Wood, Lorence & Kiehn, sp. nov. (holotype: PTBG).

#### Phenology.

To date, *Coprosma kawaikiniensis* has been observed in flower during early April, and with fruit from late August to mid-September.

#### Etymology.

The new species is named after the holotype locale, Kawaikini, the highest peak on Kaua‘i and one of the rainiest places on earth (Juvik and Juvik 1999). Literally, Kawaikini means “the multitudinous waters” in Hawaiian (Pukui et al. 1974).

#### Distribution and ecology.

The volcanic island of Kaua‘i is the oldest of the main high Hawaiian Islands (ca. 5 Ma) featuring a physical geography that is quite variable with deeply eroded drainages, well-defined canyons, and tall coastal seacliffs. It is also the most floristically rich Hawaiian Island (Wagner et al. 1990, Imada 2012) exemplified by high levels of habitat diversity and endemism, which includes ca. 244 single island vascular plant endemics (Wood 2015). Careful botanical research conducted over the last few decades by staff of the National Tropical Botanical Garden (NTBG), especially around cliffs and remote regions, has contributed 32 new published plant taxa from Kaua‘i (Imada 2012).

**Figure 2.**
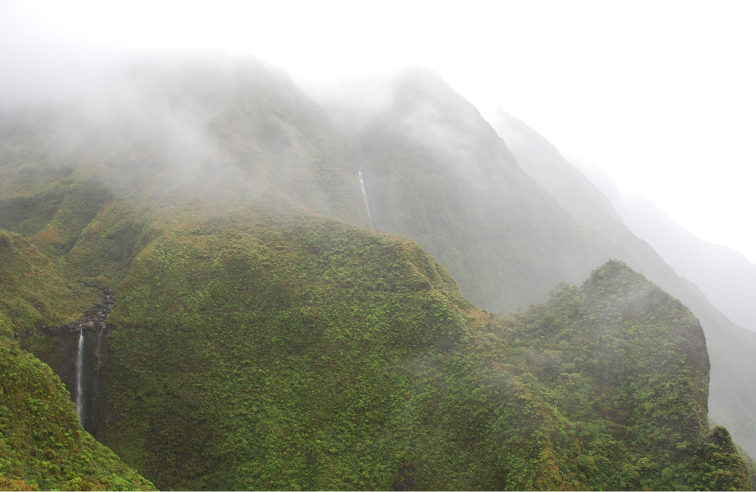
Landscape habitat where *Coprosma kawaikiniensis* was discovered below Kawaikini, Kaua‘i, showing rugged terrain and the *Dubautia*-*Sadleria* shrubland-fernland (DSSF) community.

**Figure 3.**
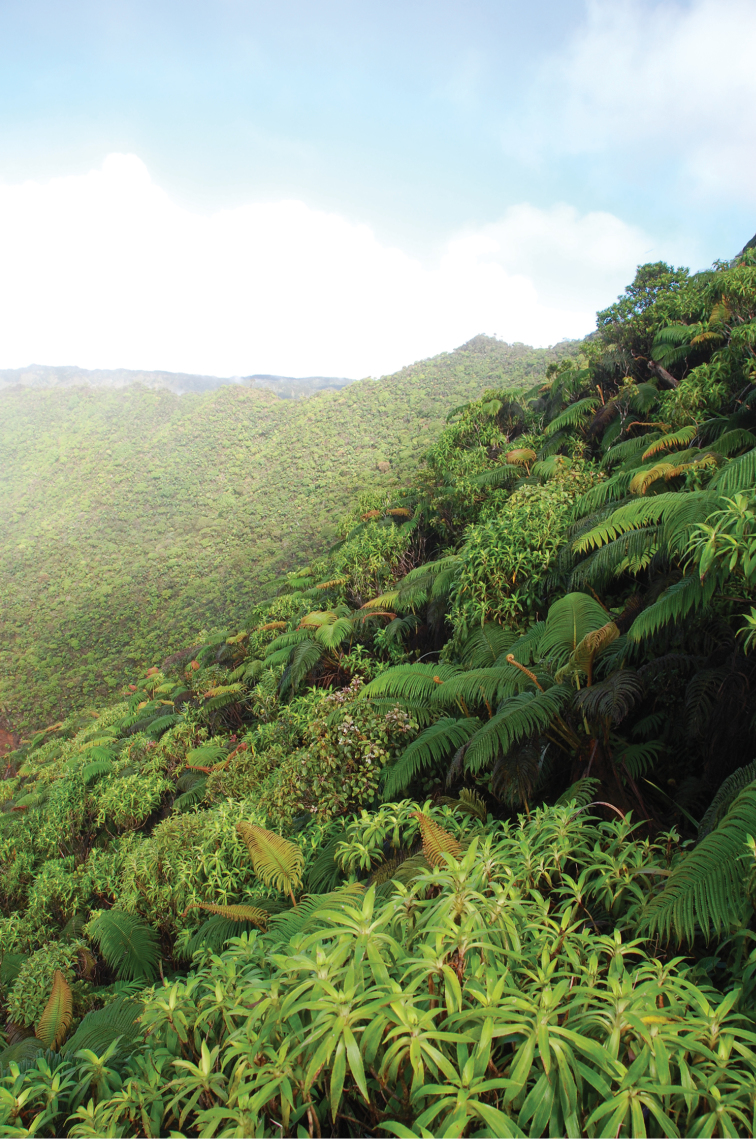
The *Dubautia*-*Sadleria* shrubland-fernland (DSSF) community below Kawaikini, Kaua‘i.

**Figure 4.**
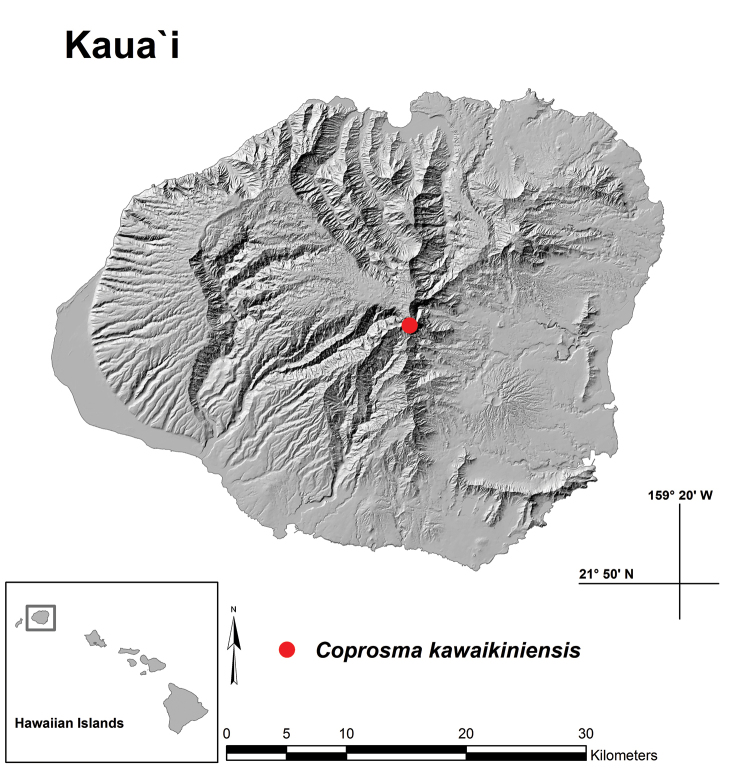
Map showing location of *Coprosma kawaikiniensis* near Kawaikini summit, Kaua‘i, Hawai‘i.

The recent discovery of *Coprosma kawaikiniensis* was made around extremely steep, narrow wind swept ridges, slopes, and boulder strewn stream banks below Kawaikini, the highest peak on Kaua‘i which summits at 1598 m elevation (Figures 2–4). This particular habitat is the remotest of Kaua‘i’s eco-regions and can be further characterized by its mist-shrouded, dark, narrow basalt canyon walls seeping with springs, and having the distinction of being one of the rainiest places in the world (Juvik and Juvik 1998). The holotype region represents a previously undescribed plant community dominated by two Hawaiian endemic genera, namely *Dubautia* Gaudich. (Asteraceae) and *Sadleria* Kaulf. (Blechnaceae), referred to as the *Dubautia*-*Sadleria* shrubland-fernland (DSSF) community (Wood 2013; Figures 2, 3). To date, fewer than 50 individuals of *Coprosma kawaikiniensis* have been documented, occurring in elevations between 1035 to 1350 m.

The DSSF community is predominantly low-statured at 1–2(–4) m tall and is composed of several species of *Dubautia* including Dubautia imbricata H.St.John subsp. acronaea G.D.Carr, Dubautia knudsenii Hillebr. subsp. nagatae (H.St.John) G.D.Carr, Dubautia laxa Hook. & Arn. subsp. hirsuta (Hillebr.) G.D.Carr, *Dubautia paleata* A.Gray, *Dubautia raillardioides* Hillebr., and on rare occasions *Dubautia pauciflorula* H.St.John & G.D.Carr. The four species of *Sadleria* that also dominate this community are *Sadleria cyatheoides* Kaulf., *Sadleria pallida* Hook. & Arn., *Sadleria souleyetiana* (Gaudich.) T.Moore, and occasionally *Sadleria squarrosa* (Gaudich.) T.Moore.

Some of the more common herbs, shrubs and trees, the latter depauperate in physical stature, of the DSSF community include Antidesma platyphyllum H.Mann var. hillebrandii Pax & Hoffm., *Astelia argyrocoma* A.Heller ex Skottsb., *Bidens forbesii* Sherff, *Broussaisia arguta* Gaudich., *Coprosma kauensis* (A.Gray) A.Heller, *Ilex anomala* Hook. & Arn., *Pritchardia hardyi* Rock, *Syzygium sandwicensis* (A.Gray) Nied., along with several to numerous species of *Cheirodendron* Nutt. ex Seem., *Cyanea* Gaudich., *Cyrtandra* J.R.Forst. & G.Forst., *Labordia* Gaudich., *Kadua* Cham. & Schltdl., *Lobelia* L., *Melicope* J.R.Forst. & G.Forst., *Metrosideros* Banks ex Gaertn., *Myrsine* L., *Peperomia* Ruiz & Pav., *Pipturus* Wedd., *Platydesma* H.Mann, *Polyscias* J.R.Forst. & G.Forst., *Psychotria* L., *Scaevola* L., and *Vaccinium* L.

Common sedges and grasses of the DSSF are *Cyperus sandwicensis* Kükenth., *Eragrostis grandis* Hillebr., Gahnia vitiensis Rendle subsp. kauaiensis (Benl) T.Koyama, *Machaerina angustifolia* T.Koyama, *Panicum lineale* H.St.John, along with several species of *Dichanthelium* (Hitchc. & Chase) Gould.

Besides the dominant *Sadleria*, there are a number of other common associated ferns found in the DSSF such as *Cibotium glaucum* (Sm.) Hook. & Arn., *Dicranopteris linearis* (Burm.f.) Underw., *Diplopterygium pinnatum* (Kunze) Nakai, *Sphenomeris chinensis* (L.) Maxon ex Kramer, as well as several species of *Adenophorus* Gaudich., *Asplenium* L., *Dryopteris* Adans., *Elaphoglossum* Schott, and *Hymenophyllum* Sm.

Plant communities of the nearby surrounding region are all montane windward wet habitat associations. Open bogs occur in scattered locations along the main upper headwater ridge line and are dominated by genera such as *Metrosideros*, *Rhynchospora* Willd., *Dichanthelium*, *Gahnia* J.R.Forst. & G.Forst., *Machaerina* Vahl, *Plantago* L., *Viola* L., and *Oreobolus* R.Br. Several very rare species associated with bogs and bog margins were also discovered, namely *Keysseria helenae* (C.N.Forbes & Lydgate) Cabrera and *Lysimachia venosa* (Wawra) H.St.John, a member of the Primulaceae family undocumented since 1911 (Wood 2013). Occasional stands of *Metrosideros*-*Cheirodendron* forest with low-statured canopies of 5–7 m also occur in a mosaic of random patches surrounding the DSSF community, and *Coprosma kawaikiniensis* has also been observed ranging into this habitat. These forests are usually dominated by a significant diversity of mixed understory associates very similar to that of the DSSF. The riparian regions that dissect these relic *Metrosideros*-*Cheirodendron* forests and form the headwater drainages that feed the respective lower elevation valleys retain a flourishing treasure trove of rare plant taxa along nearby stream banks, including endangered species of *Cyanea*, *Cyrtandra*, *Hesperomannia* A.Gray, *Isodendrion* A.Gray, *Labordia*, *Melicope*, *Phyllostegia* Benth., *Platydesma*, and *Polyscias*.

Vegetation cover on several nearby steep slopes is composed of just a few species of matting ferns (i.e., *Dicranopteris linearis*, *Diplopterygium pinnatum*, and *Sticherus owhyhensis* (Hook.) Ching) and may be the resulting succession of past landslides. Vertical wet cliff communities of sedges, herbs, and ferns, accentuated with numerous waterfalls form the prevalent back-drop that tower over and surround these habitats below Kawaikini (Wood 2014) (Figure 2).

The holotype region of *Coprosma kawaikiniensis* has very few non-native plants and animals, however, *Coprosma kawaikiniensis* is susceptible to catastrophic extinction through environmental events such as hurricanes, landslides, and flash floods in addition to the incursion of threats from nearby regions. Native habitats in the adjacent lower elevation regions, especially below 730 m, are intensely threatened by habitat degradation from feral goats (*Capra hircus* L.) and pigs (*Sus scrofa* L.), predation of seeds by rats (*Rattus rattus* L. and *Rattus exulans* Peale), and competition with non-native plant taxa, especially *Axonopus fissifolius* (Raddi) Kuhlm., *Blechnum appendiculatum* Willd., *Buddleia asiatica* Lour., *Clidemia hirta* (L.) D.Don, *Cyperus meyenianus* Kunth, *Erigeron karvinskianus* DC., *Juncus planifolius* R.Br., *Melastoma septemnervium* Lour., *Paspalum conjugatum* P.J.Bergius, *Psidium cattleianum* Sabine, *Rhodomyrtus tomentosa* (Aiton) Hassk., *Rubus rosifolius* Sm., *Sacciolepis indica* (L.) Chase, and *Sphaeropteris cooperi* (Hook. ex R.Muell.) R.M.Tryon. These highly invasive weeds possess the ability to spread rapidly (Smith 1995) and have begun to ingress into the upper watershed habitats of *Coprosma kawaikiniensis* (Wood 2014).

#### Conservation status.

*IUCN Red List Category*. When evaluated using the World Conservation Union (IUCN) criteria for endangerment (IUCN 2001), *Coprosma kawaikiniensis* falls into the Critically Endangered (CR) category, which designates this species as facing a very high risk of extinction in the wild. Our formal evaluation can be summarized by the following IUCN hierarchical alphanumeric numbering system of criteria and subcriteria: CR B1ab(i,ii,iii,v)+2ab(i,ii,iii,v); C2a(ii); D; which reflects a severely limited Extent of Occurrence (EOO) and Area of Occupancy (AOO) of less than 3 km^2^ and a population of less than 50 individuals.

#### Discussion.

**Figure 5.**
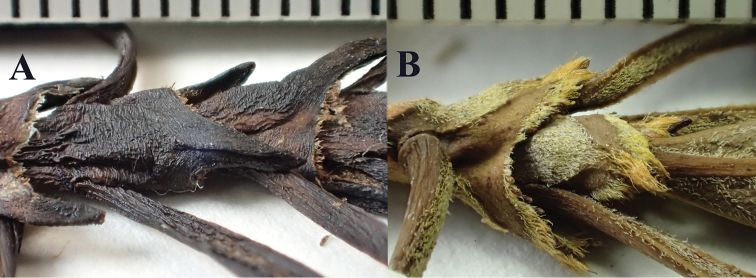
Stipules and petiole bases of *Coprosma kawaikiniensis* (**A** *Wood 3539*, paratype PTBG) and *Coprosma kauensis* (**B** *Perlman 18645*, PTBG). Scale bars in mm.

All species of *Coprosma* from Kaua‘i have stipules shorter than 4.5 mm except for *Coprosma kawaikiniensis* and *Coprosma kauensis*. The features distinguishing those two species are the number of the flowers per partial inflorescence [6–8, sometimes with an additional pair of 3–5 flowered cymules in *Coprosma kawaikiniensis* vs. 3(–5) flowered in *Coprosma kauensis*], the length of the calyx in staminate flowers (1.8–3.2 mm in *Coprosma kawaikiniensis* vs. 0.4–0.6 mm in *Coprosma kauensis*), the length of the staminate peduncle (20–28 mm in *Coprosma kawaikiniensis* vs. 4–8 mm in *Coprosma kauensis*), the pubescence of the stipules (externally glabrous in *Coprosma kawaikiniensis* vs. densely strigose on both surfaces in *Coprosma kauensis*), and the number and form of the stipular appendage(s) [only 1 terminal claw-like appendage and occasionally a few short, rounded appendages or callous protuberances 0.1–0.2 mm long c. equaling the ciliate marginal hairs in *Coprosma kawaikiniensis*, vs. (3–)5–7(–8) pairs of thickly ovoid to digitate, shiny dark brown-black marginal appendages 0.3–0.5 mm long in *Coprosma kauensis*] (Figure 5).

Species of *Coprosma* that have stipules of 4.5 mm or greater from the remaining Hawaiian Islands include: *Coprosma longifolia* A.Gray, *Coprosma ternata* W.R.B.Oliv., *Coprosma rhynchocarpa* A. Gray, *Coprosma montana* Hillebr., and *Coprosma ochracea* W.R.B.Oliv. *Coprosma kawaikiniensis* differs from *Coprosma longifolia* and *Coprosma ternata* in having opposite vs. usually ternate leaves (with only occasionally opposite leaves on some stems). *Coprosma kawaikiniensis* has peduncles 20–28 mm long vs. 2.5–16 mm long in *Coprosma rhynchocarpa* and only 0–1(–4) mm long in *Coprosma ochracea*. The new species differs from *Coprosma montana* in having 6–8 flowers per cluster, sometimes with an additional pair of 3–5 flower cymules vs. 1–2 per cluster. In terms of morphology, *Coprosma kawaikiniensis* most closely resembles *Coprosma longifolia* (Oahu), but differs from the latter in having opposite vs. usually ternate leaves, stipules with a shorter sheath (1–)2–4 mm long, and free apical portion (3–)4–6 mm long terminated by a thickly carinate-subulate apex with a claw-like appendage, vs. sheath 7–9 mm long and free apical part 2–3 mm long with short-attenuate, acute tip.

The following couplets can be inserted into the existing key to Hawaiian *Coprosma* by Wagner et al. (1990: 1123) to accommodate *Coprosma kawaikiniensis*.

**Table:** 

4(2)	stipules 4.5–11(–15) mm long	**5**
4	stipules 1.5–4 mm long	**9**
5(4)	Staminate calyx 1.8–3.2 mm long; staminate corolla lobes ca. 3.5–6 mm long	**5a**
5	Staminate calyx 0.4–2 mm long; staminate corolla lobes 2–4.5 mm long	**6**
5a(5)	Staminate calyx ca. 3 mm long; staminate corolla lobes ca. 5–6 mm long; pistillate calyx urceolate, 2–4 mm long, enlarging to 5–10 mm long in fruit; H	**11. *Coprosma rhynchocarpa***
	Staminate calyx ca. 1.8–3.2 mm long; staminate corolla lobes 3.5–4 mm long; pistillate calyx unknown, but not enlarging in fruit; K	**14. *Coprosma kawaikiniensis***
6(5)	Stipule margins without appendages except 1 at apex, short-ciliate, base puberulent; flowers 1–2 per cluster; usually subalpine, above 1,830 m, EM, H.	**8. *Coprosma montana***
(6)	Stipule margins with conspicuous appendages, but often obscured by pubescence in *Coprosma ochracea*, and sparsely ciliate, pilose, or long-hirsute to short-hirsute, base strigose, appressed or spreading hirsute, short-hirsute, pilose or glabrous; flowers 3–6 per cluster	**7**
7(6)	Peduncles 0–1 mm long	**9. *Coprosma ochracea***
7	Peduncles 4–28 mm long	**7a**
7a(7)	Staminate calyx 1.8–3.2 mm long	**14. *Coprosma kawaikiniensis***
7a	Staminate calyx 0.4–1 mm long	**8**

#### Additional specimens examined

**(paratypes).** United States of America. Hawai‘i. Kaua‘i: Lihu‘e District, ridge running south of Kawaikini, above Iliiliula and Ioli drainage, 1035 m elev., 19 Sep 1994, *K.R. Wood, P. Wood, S. Perlman 3539*, (BISH, PTBG, US); ridge just south below Kawaikini, 1130-1350 m elev., 03 Apr 2013, *K.R. Wood 15460* (BISH, PTBG, US); loc. cit., 03 Apr 2013, *K.R. Wood 15463* (PTBG, US, WU).

## Supplementary Material

XML Treatment for Coprosma kawaikiniensis
